# Sirtuin 6 inhibits group 3 innate lymphoid cell function and gut immunity by suppressing IL-22 production

**DOI:** 10.3389/fimmu.2024.1402834

**Published:** 2024-08-26

**Authors:** Xiaohui Su, Linfeng Zhao, Huasheng Zhang, Dongdi Wang, Jiping Sun, Lei Shen

**Affiliations:** ^1^ Center for Immune-Related Diseases at Shanghai Institute of Immunology, Ruijin Hospital, Shanghai Jiao Tong University School of Medicine, Shanghai, China; ^2^ Department of Immunology and Microbiology, Key Laboratory of Cell Differentiation and Apoptosis of Chinese Ministry of Education, Shanghai Jiao Tong University School of Medicine, Shanghai, China; ^3^ Shanghai Key Laboratory of Tumor Microenvironment and Inflammation, Shanghai Jiao Tong University School of Medicine, Shanghai, China

**Keywords:** sirtuin 6, group 3 innate lymphoid cells, IL-22, gut immunity, inflammation

## Abstract

**Introduction:**

Group 3 innate lymphoid cells (ILC3s) are enriched in the intestinal mucosa and play important roles in host defense against infection and inflammatory diseases. Sirtuin 6 (SIRT6) is a nicotinamide adenine dinucleotide (NAD+)- dependent deacetylase and has been shown to control intestinal epithelial cell differentiation and survival. However, the role of SIRT6 in ILC3s remains unknown.

**Methods:**

To investigate the role of SIRT6 in gut ILC3s, we generated SIRT6 conditional knockout mice by crossing Rorccre and Sirt6flox/flox mice. Cell number and cytokine production was examined using flow cytometry. Citrobacter rodentium infection and dextran sodium sulfate-induced colitis models were used to determine the role of SIRT6 in gut defense. RT-qPCR, flow cytometry and immunohistochemistry were used to assess the intestinal inflammatory responses.

**Results:**

Here we show that SIRT6 inhibits IL-22 expression in intestinal ILC3s in a cell-intrinsic manner. Deletion of SIRT6 in ILC3s does not affect the cell numbers of total ILC3s and subsets, but results in increased IL-22 production. Furthermore, ablation of SIRT6 in ILC3s protects mice against Citrobacter rodentium infection and dextran sodium sulfate-induced colitis. Our results suggest that SIRT6 may play a role in ILC3 function by regulating gut immune responses against bacterial infection and inflammation.

**Discussion:**

Our finding provided insight into the relation of epigenetic regulators with IL-22 production and supplied a new perspective for a potential strategy against inflammatory bowel disease.

## 1 Introduction

Innate lymphoid cells (ILCs) are lymphoid-derived innate cells that lack recombination activating gene (RAG)-dependent rearranged antigen receptors (1). Based on the expression of transcription factor and cytokines secretion profile during ILC development, ILCs can be divided into three groups: group 1 ILCs (ILC1s) express T-bet and produce interferon-γ (IFN-γ), group 2 ILCs (ILC2s) require GATA3 and produce IL-5 and IL-13, and group 3 ILCs (ILC3s) are defined by the expression of RAR-related orphan receptor gamma t (RORγt) and production of IL-22 and/or IL-17 (2–5).

ILC3s are abundantly localized in the lamina propria of the intestine, where they play important roles in tissue homeostasis, host defense during bacterial infection, and tissue repair during intestinal inflammation (6–8). ILC3 can secrete high levels of cytokine IL-22. IL-22 is an important anti-infective medium, which can stimulate intestinal epithelial cells (IEC) to secrete antimicrobial peptides and play a role in clearing intestinal pathogenic microorganisms (9–11). IL-22 also promotes intestinal epithelial cell fucosylation through the induction of the fucosyltransferase Fut2 to protect from infection (12, 13). During inflammatory tissue damage, such as inflammatory bowel disease or graft-versus-host disease (GVHD), IL-22 is important for maintaining intestinal epithelial integrity by inducing epithelial cell proliferation and intestinal stem cell differentiation (14–17). Emerging studies indicate that disruption of the ILC3 compartment has been associated with inflammatory bowel disease and intestinal infection (18, 19). IL-22-producing ILC3s were decreased in the intestine of Crohn’s patients (20, 21). Deletion of IL-22 in ILCs resulted in increased susceptibility to *Citrobacter rodentium* (*C. rodentium)* infection (18). Therefore, understanding the molecular mechanisms responsible for ILC3 activation and IL-22 production is critical for modulating intestinal immune responses during bacterial infection and inflammatory disease.

The Sirtuin family members (SIRTs) are evolutionarily conserved NAD^+^-dependent enzymes (22, 23). SIRT6 is a nuclear and chromatin-bound protein that has been implicated in DNA repair, genome stability, metabolism, and inflammation (24–26). Studies have shown that SIRT6 plays a protective role in colitis (27). Deletion of *Sirt6* in IEC resulted in increased susceptibility to dextran sodium sulfate (DSS)-induced colitis in mice. By contrast, overexpressing *Sirt6* improved DSS-induced colitis. A recent study reveals that IEC *Sirt6* deletion can also cause impaired tuft cell development and type 2 immunity in response to helminth infection (28). Mice with airway epithelial cell-specific deletion of *Sirt6* are protected against allergen-induced airway inflammation (29). However, the role of SIRT6 in regulating ILC3 function and intestinal homeostasis has not been investigated. Using *Sirt6^ΔRorc^* mice of both the wild-type and *Rag1^−/−^* background, we show that SIRT6 inhibits IL-22 production and ILC3 function in a cell-intrinsic manner. Our study provides new insights into the regulation of ILC3 activation and intestinal immunity.

## 2 Materials and methods

### 2.1 Mice

*Sirt6^fl/fl^* mice and *Rag1^-/-^* mice were purchased from the Jackson Laboratory. *Rorc^Cre^* mice was provided by Ju Qiu from the Shanghai Institute of Nutrition and Health in the Chinese Academy of Sciences which was purchased from the Jackson Laboratory. The mice used in this study were C57BL/6 background. *Sirt6^fl/fl^* mice possess loxP sites flanking exons 2-3 of the *Sirt6* targeted gene. *Rorc^Cre^* mice express cre recombinase under the control of the mouse *Rorc* (RAR-related orphan receptor gamma; also called RORγt) promoter. *Rag1^-/-^* mice produce no mature T cells or B cells. *Sirt6^fl/fl^* and *Rorc^Cre^* mice were crossed to obtain a *Sirt6^ΔRorc^* strain. *Sirt6^ΔRorc^* and *Rag1^-/-^* mice were crossed to obtain a *Rag1^-/-^Sirt6^ΔRorc^* strain. All mice were bred and maintained at accredited animal facilities under specific-pathogen-free conditions in individually ventilated cages on a strict 12-hour day–night cycle with a regular chow diet. Mice used in this study were 6–8 weeks old and sex-matched, unless otherwise indicated in the text. Mice were maintained at Shanghai Jiao Tong University School of Medicine. All animal experiments were performed in compliance with the Guide for the Care and Use of Laboratory Animals, with the protocols approved by the Institutional Animal Care and Use Committee of Shanghai, China [SYXK(SH)2023-0041].

### 2.2 Isolation of intestinal lamina propria lymphocytes

LPLs were purified as previously described (30). Briefly, mouse intestines (with Peyer’s patches removed) were opened lengthwise and cut into 2 cm pieces. Intestinal pieces were washed with 1 mM DTT/PBS on a rocking platform for 10 min and then intensely shaken for 2 min. Repeat the last operation with 30mM EDTA/PBS. The remaining intestinal pieces were further chopped into 5 mm pieces and digested with RPMI media containing 100U/ml of Collagenase VIII (Sigma-Aldrich, C-2139) and 150ug/ml of DNase I (Sigma-Aldrich, DN25) at 37 °C in a 5% CO_2_ incubator for 90min. The preparations were filtered with a 70µm cell strainer and subjected to density gradient centrifugation using 40% and 80% Percoll solutions (GE Healthcare) at 2500rpm for 20min. Lymphocytes were collected from the interface and washed once.

### 2.3 *C. rodentium* infection

*C. rodentium* strain DBS100 was cultured overnight in Luria-Bertani (LB) broth shaking at 37°C. Adult male *Sirt6^fl/fl^* and *Sirt6^ΔRorc^* littermates or *Rag1* ^-/-^ *Sirt6^fl/fl^* and *Rag1* ^-/-^ *Sirt6^ΔRorc^* littermates (6-8 weeks) were orally gavaged with 10^10^ colony-forming units (CFU) of *C. rodentium* in 0.2 mL PBS. At five days post-inoculation, Feces were collected, weighed, homogenized, serially diluted and plated to determine the CFU. At six days post-inoculation, mice were sacrificed, and the colons were collected and divided into 3 pieces. One piece was flash-frozen for RNA analysis and the second was processed for flow cytometry analysis, and the third was fixed with 4% paraformaldehyde for histological analysis.

### 2.4 DSS-induced colitis

*Sirt6^fl/fl^* and *Sirt6^ΔRorc^* mice at the age of 6-8 weeks were oral administration of 2.5% DSS (MP Biomedical) in the drinking water for 7 days, followed by 3 days of normal drinking water. Body weight, the presence of occult or gross blood per rectum, and stool consistency were determined daily. Changes in body weight were indicated as loss of baseline body weight as a percentage. The scoring system described by Kim et al. was used to determine the disease activity index (DAI) (31). The DAI is the combined score of weight loss compared to initial weight, stool consistency, and bleeding. Scores are defined as follows: weight loss: 0 (no loss), 1 (1-5%), 2 (5-10%), 3 (10-20%), and 4 (>20%); stool consistency: 0 (normal), 1 (soft), 2(very soft) and 3 (Watery); and bleeding: 0 (no blood), 1 (hemoccult positive), 2 (hemoccult positive and visual pellet bleeding), and 3 (gross bleeding, blood around anus). DAI can be scored daily during the duration of the DSS treatment. Colons were collected and divided into 2 pieces at 10 days after DSS treatment. One piece was processed for flow cytometry analysis, and another one was fixed with 4% paraformaldehyde for histological analysis.

### 2.5 Flow cytometry and cell sorting

Cells were resuspended in PBS and stained with fixable viability dye (BD Biosciences) at room temperature for 15 min to exclude dead cells. After washing with PBS, Cells were resuspended in FACS buffer and incubated with Fc Block (Biolegend,1:50) for 5 min at 4°C to block Fc receptors. For surface staining, cells were incubated with antibody cocktails for 30 minutes at 4°C in the dark. For intracellular cytokine staining, cells were stimulated with 50ng/ml of PMA (Sigma-Aldrich) and 500ng/ml of ionomycin (ENZO) or 10ng/ml of IL-23 for 4 hours and 2 µg/ml of Brefeldin A (Biolegend) was added for the last 2 hours. After surface staining, cells were fixed and permeabilized using the Foxp3/Transcription Factor Staining Buffer Set (eBioscience), and stained with antibody for 45 minutes at 4°C in the dark. For cell sorting, ILC3s were identified as Lin^-^CD90^hi^CD45^int^ live lymphocytes using a FACSAriaIII (BD Biosciences) (32–35). Lineage markers include CD3, Gr-1, B220, CD11b, and CD11c. All reagents used were listed in **Supplementary Table S1** and all antibodies used were listed in **Supplementary Table S2**. Flow cytometry data were acquired using LSRFortessaX20 (BD Biosciences) and analyzed with Flowjo software (version 10).

### 2.6 Cell culture

Sorted ILC3s were cultured in 96-well flat-bottom plates in RPMI 1640 complete medium containing 10% fetal bovine serum, 2mM L-glutamine, 50μM β-mercaptoethanol, 1% MEM NEAA, 1% penicillin/streptomycin (all from Gibco or Sigma-Aldrich), in the presence of 10ng/ml IL-7 (Peprotech) (36).

The parental MNK cell lines are derived from NKR^-^P1B^+^ fetal thymocytes. Among them, MNK3 is an IL-7-responsive subline that expresses Rorγt and produces high levels of IL-22 in response to IL-23 and IL-1β stimulation (37). The MNK3 cell line was previously described as an *in vitro* system to study ILC3 functionality (37). MNK3 cells were cultured in Dulbecco’s modified Eagle’s medium-high glucose medium with 10% fetal bovine serum, 2mM GlutaMAX, 1mM sodium pyruvate, 55μM β-mercaptoethanol, 10mM HEPES, 100μg/ml streptomycin, and 1% penicillin/streptomycin (all from Gibco or Sigma-Aldrich). MNK3 cells were cultured in the presence of 10ng/ ml mouse IL-7 (Peprotech) and 10ng/ml mouse IL-15 (Peprotech).

Both ILC3s and MNK3 were cultured at 37°C in 5% CO2 humidified air.

For inhibitor treatment, sorted ILC3s and MNK3 were treated with OSS-128167 (100μM, Selleck) for 18 hours *in vitro* and then stimulated with IL-23 at 10ng/ml for another 4hours.The percentage of IL-22^+^ ILC3s or MNK3 was determined by flow cytometry.

### 2.7 Detection of mRNA by qRT-PCR

Total RNA was isolated with Trizol (Invitrogen). RNA concentration was detected by Nanodrop Spectrophotometer (Nanodrop Technologies). cDNA was synthesized using PrimeScript™ RT reagent Kit (Takara). Quantitative real-time reverse transcription qRT-PCR was performed using SYBR™ Select Master Mix (Applied Biosystems™) on a ViiA7 Real-Time PCR System. Results were normalized as *hprt* through the ΔΔCt method. Primer sequences were shown in the **Supplementary Table S3**.

### 2.8 Histology

Flush the colon with 10 mL of ice-cold phosphate-buffered saline (PBS) with a 10 mL syringe equipped with a gavage needle to remove the feces and blood until the eluate is completely clear. Cut it open longitudinally. Place the intestinal segment with the luminal side facing upward. Proceed with Swiss rolling the colon (38). Then fix the tissue with 4% paraformaldehyde overnight at 4°C for paraffin embedding. For staining, cut 4um thick sections using a microtome. The sections were stained with hematoxylin staining solution for 5 min, washed with tap water, differentiated with differentiation solution, and returned to a blue solution. The slices were dehydrated with 85% and 95% gradient alcohol for 5 min respectively, and stained with eosin staining solution for 5 min. Sections were observed and photographed using the OLYMPUS BX53 system.

### 2.9 Statistical analysis

Statistical analyses were performed using the GraphPad Prism 8.0 program. Unless otherwise noted, the Kolmogorov-Smirnov test was performed for normality tests. Statistical significance was tested using two-tailed unpaired Student’s t-test as parametric tests or Mann-Whitney test as nonparametric tests. For two independent variables, the differences were measured by two-way ANOVA. Data from these experiments are presented as the mean values ± SEM. *P* values are noted in each figure.

## 3 Results

### 3.1 Ablation of SIRT6 in ILC3s enhances IL-22 production in steady state

**Figure 1:**
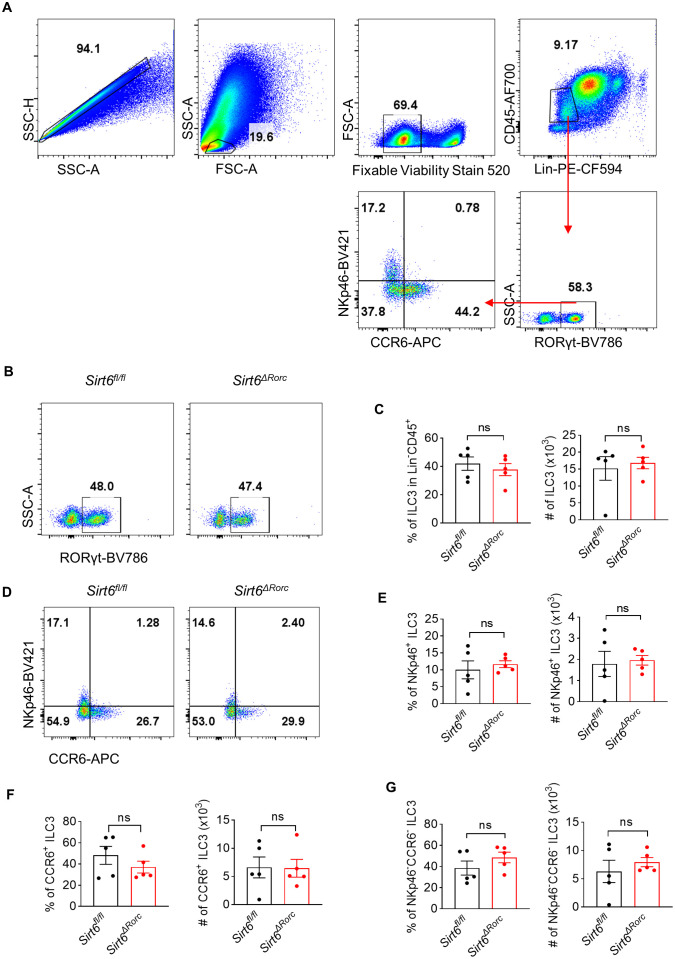
SIRT6 is not required for ILC3 development. **(A)** Gating strategy of ILC3s (Lin^-^ CD45^+^RORγt^+^) in *Sirt6^fl/fl^* and *Sirt6^ΔRorc^* mice. **(B)** Representative flow cytometry plots for ILC3s gated on Lin^-^CD45^+^. **(C)** Percentage and absolute number of total ILC3s (RORγt^+^ among Lin^-^CD45^+^) in *Sirt6^fl/fl^* and *Sirt6^ΔRorc^* mice (n=5/group). **(D)** Representative flow cytometry plots for ILC3 subsets gated on Lin^-^CD45^+^RORγt^+^. **(E-G)** Frequencies and cell counts of NKp46^+^ ILC3s **(E)**, CCR6^+^ ILC3s **(F)**, NKp46^-^CCR6^-^ ILC3s **(G)** in *Sirt6^fl/fl^* and *Sirt6^ΔRorc^* mice (n=5/group). Each symbol represents an individual mouse **(C, E-G)**. Data are representative of 2 independent experiments and are presented as mean ± SEM. For statistical analysis, the following tests were used. C-left, E-G, two-tailed unpaired Student’s t-test. C-right, Mann-Whitney test.

**Figure 2:**
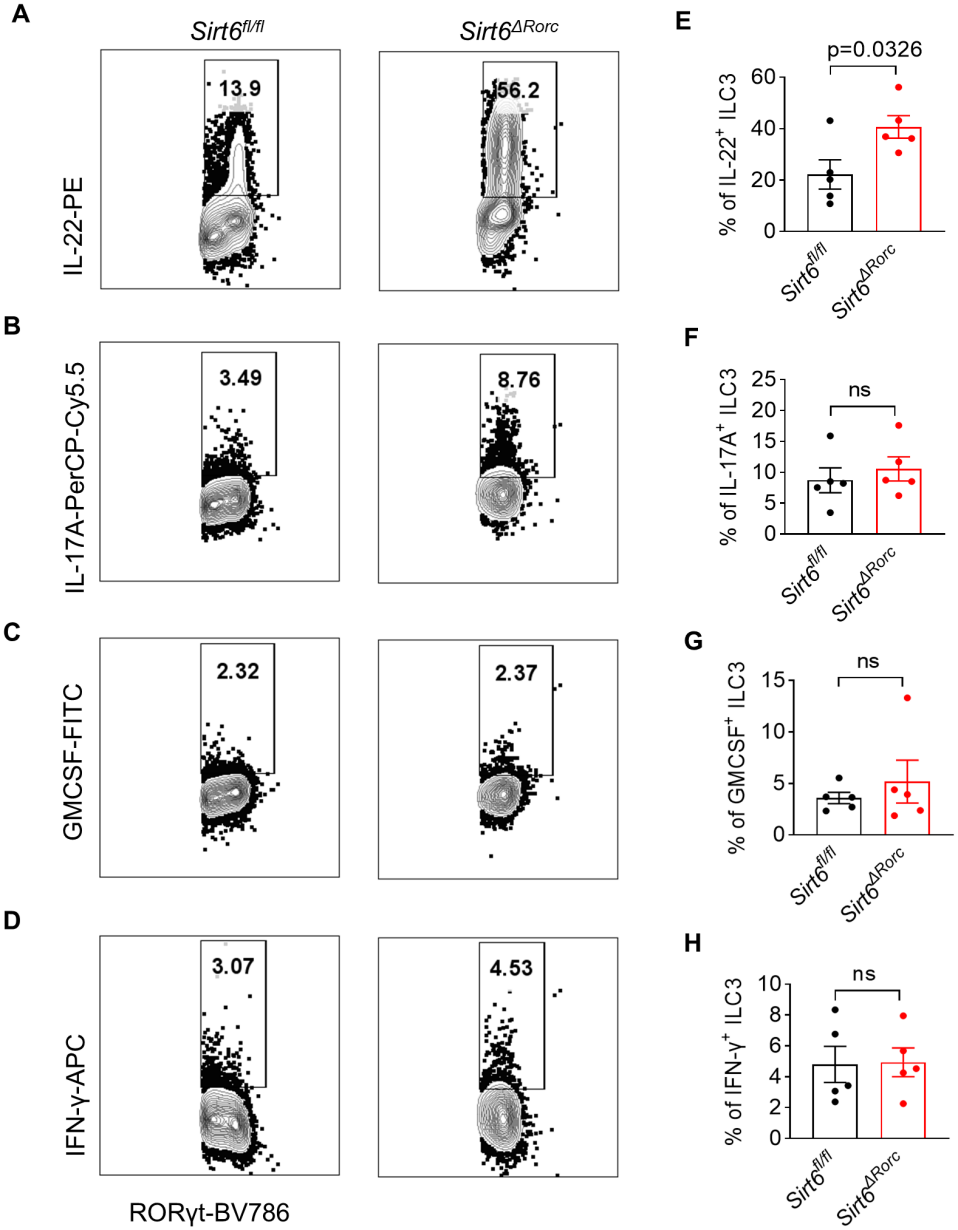
SIRT6 deletion in ILC3s promotes IL-22 production in response to IL-23. **(A-H)** LPLs from *Sirt6^fl/fl^* and *Sirt6^ΔRorc^* mice were treated with 10ng/ml of IL-23 for 4h. **(A-D)** Representative flow cytometry plots for IL-22 **(A)**, IL-17A **(B)**, GMCSF **(C)**, and IFN-γ **(D)** produced by ILC3s gated on Lin^-^CD45^+^RORγt^+^. **(E-H)** Frequencies of IL-22^+^ ILC3s **(E)**, IL-17A^+^ ILC3s **(F)**, GMCSF^+^ ILC3s **(G)**, IFN-γ^+^ ILC3s **(H)**. n=5/group **(E-H)**. Each symbol represents an individual mouse **(E-H)**. Data are representative of 2 independent experiments and are presented as mean ± SEM. For statistical analysis, the following tests were used. E,F,H, two-tailed unpaired Student’s t-test. G, Mann-Whitney test.

To investigate the role of SIRT6 in gut ILC3s, we crossed *Sirt6^fl/fl^* mice with *Rorc^Cre^* mice to knock out SIRT6 in ILC3s (*Sirt6^ΔRorc^*) (**Supplementary Figure S1**). Genotyping of mice was performed by PCR (**Supplementary Figure S1A**). The efficiency of SIRT6 deletion in ILC3s exceeded 75% (**Supplementary Figure S1B**). We found that deletion of SIRT6 did not affect body weight and colon length in mice (**Supplementary Figures S1C, D**). We then examined the effect of SIRT6 on the ILC3 compartment in the small intestine. The Gating strategy of ILC3 is shown in **Figure 1A** as previously described (6, 32). Neither the frequency nor the absolute number of ILC3s was affected in *Sirt6^ΔRorc^* mice compared with *Sirt6^fl/fl^* control mice (**Figures 1B, C**). The composition of ILC3 subsets, including NKp46^+^, CCR6^+^, and CCR6^−^NKp46^−^ had no change in *Sirt6^ΔRorc^* mice as well (**Figures 1D-G**). Given that ILC3s exert immune function through the production of effector cytokines, we examined the expression of IL-22, IL-17A, GMCSF, and IFN-γ by ILC3s. Intestinal ILC3s were treated with activating cytokine IL-23. We observed that SIRT6-deficient ILC3s produced more IL-22, while IL-17A, GMCSF, and IFN-γ were unaffected (**Figures 2A-H**). Together, these data suggest that SIRT6 suppresses IL-22 production by ILC3s in the gut.

### 3.2 SIRT6 ablation in ILC3s protects mice from *C. rodentium* infection

**Figure 3:**
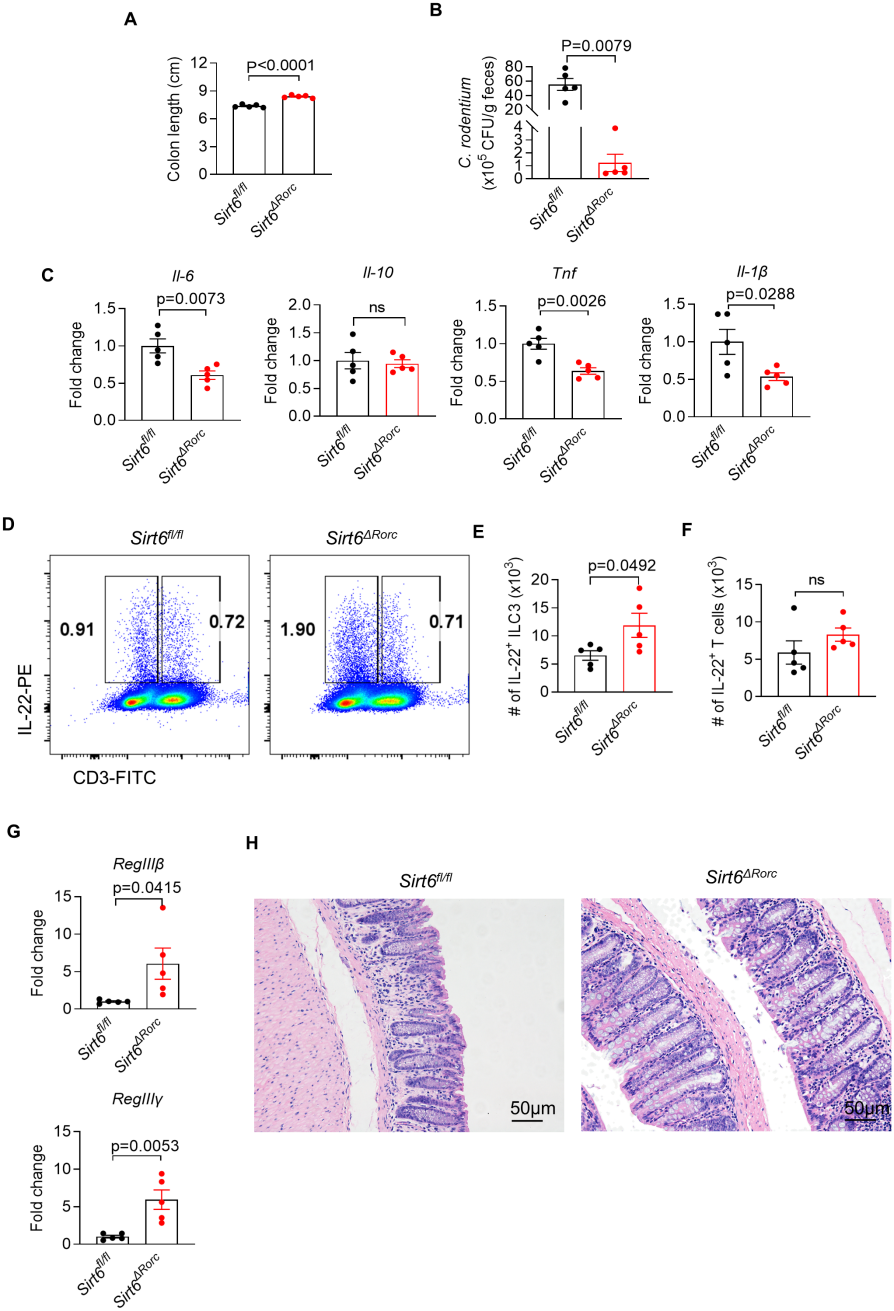
Loss of SIRT6 enhances ILC3 mediated mucosal defense against bacterial infection. **(A-H)** *Sirt6^fl/fl^* and *Sirt6^ΔRorc^* mice were orally gavaged with 10^10^ CFU of *C. rodentium*. Large intestine tissues were collected 6 days after infection. **(A)** Length of the colon from *Sirt6^fl/fl^* and *Sirt6^ΔRorc^* mice (n=5/group). **(B)** The amount of *C. rodentium* in *Sirt6^fl/fl^* and *Sirt6^ΔRorc^* mice was measured (n=5/group). Bacterial counts were normalized to CFU per gram of feces. **(C)** mRNA expression of pro-inflammatory cytokines in the large intestine from *Sirt6^fl/fl^* and *Sirt6^ΔRorc^* mice (n=5/group). **(D)** Representative flow cytometry plot for IL-22 produced by ILC3s and T cells. Cells were gated on Gr-1^-^B220^-^CD11b^-^CD11c^-^CD45^+^ live lymphocytes. **(E, F)** Absolute numbers of IL-22 producing ILC3s **(E)** and T cells **(F)** in large intestine lamina propria of *Sirt6^fl/fl^* and *Sirt6^ΔRorc^* mice (n=5/group). **(G)** Expression of *RegIIIβ, RegIIIγ* in large intestine from *Sirt6^fl/fl^* and *Sirt6^ΔRorc^* mice (n=5/group). **(H)** Representative image of H&E staining of colon sections from *C.rodentium* infected mice at day 6. The scale bar is 50μm. Each symbol represents an individual mouse **(A-C, E-G)**. Data are representative of 2 independent experiments and are presented as mean ± SEM. For statistical analysis, the following tests were used. A,C,E-G, two-tailed unpaired Student’s t-test. B, Mann-Whitney test.

We next determined the role of SIRT6 in ILC3s during *C. rodentium* infection. Mice were challenged by oral gavage with 10^10^ CFU of *C. rodentium*. Compared to *Sirt6^fl/fl^* littermate controls, *Sirt6^ΔRorc^* mice controlled bacterial infection more efficiently, as indicated by longer colon length and reduced bacterial load (CFU counts) in feces (**Figures 3A, B**). Consistently, *Sirt6^ΔRorc^* mice exhibited reduced tissue inflammation characterized by decreased expression of pro-inflammatory cytokines including *Il-6, Tnf, and Il-1β*, compared to the *Sirt6^fl/fl^* littermate controls (**Figure 3C**). As IL-22 has a crucial role in the early phase of host defense against *C. rodentium*, we examined the expression of IL-22 in ILC3s and T cells in the large intestine by flow cytometry (**Figures 3D-F**). As expected, infection with *C. rodentium* induced the expression of IL-22 in ILC3s. On day 6 post-infection, ILC3s from *Sirt6^ΔRorc^* mice produced more IL-22 than *Sirt6^fl/fl^* littermate controls (**Figures 3D,E**). Since SIRT6 was deleted in both ILC3s and T cells in *Sirt6^ΔRorc^* mice, IL-22 production by T cells was assessed during *C. rodentium* infection. We did not observe significant differences in IL-22-expressing T cells between *Sirt6^fl/fl^* and *Sirt6^ΔRorc^* mice (**Figures 3D, F**). Since IL-22 protects epithelial function by stimulating the secretion of antimicrobial peptides, we next analyzed the expression of the genes encoding the antimicrobial peptides. The expression of *RegIIIβ* and *RegIIIγ* were increased in the *Sirt6^ΔRorc^* mice (**Figure 3G**). Consistently, *Sirt6^ΔRorc^* mice exhibited reduced tissue inflammation, as indicated by less immune cell infiltration in H&E staining (**Figure 3H**). Thus, SIRT6 plays a critical role in regulating ILC3 function during *C. rodentium* infection.

### 3.3 *Sirt6* regulates ILC3 function and gut immunity in a cell-intrinsic manner

Since Rorc-driven Cre recombinase is expressed in both T cells and ILC3s, we investigated whether T cells were affected in *Sirt6^ΔRorc^* mice. *Sirt6^ΔRorc^* mice did not show any defects in the small intestine T cells. Neither the frequency nor the absolute number of RORγt^+^ T cells (gating on Gr-1^-^B220^-^CD11b^-^CD11c^-^CD45^+^CD3^+^RORγt^+^) was affected in *Sirt6^ΔRorc^* mice compared with *Sirt6^fl/fl^* control mice (**Supplementary Figures S2A, B**). Furthermore, the composition of RORγt^+^ T cells subsets, including RORγt^+^ Th17, RORγt^+^ Treg, and RORγt^+^CD4^-^ T cells had no change in *Sirt6^ΔRorc^* mice (**Supplementary Figures S2A, C**). LPLs from the small intestine were stimulated with PMA/ionomycin and followed by intracellular staining for cytokines production. The expression of IFN-γ, IL-4, and IL-17A by CD3^+^ T cells showed no differences between *Sirt6^fl/fl^* and *Sirt6^ΔRorc^* mice (**Supplementary Figures S3A-F**). By contrast, IL-22-expressing ILC3s were largely increased despite normal IL-22 production by T cells in *Sirt6^ΔRorc^* mice (**Supplementary Figures S3G-I**). Thus, the T cell compartment in the gut was not affected in *Sirt6^ΔRorc^* mice.

**Figure 4:**
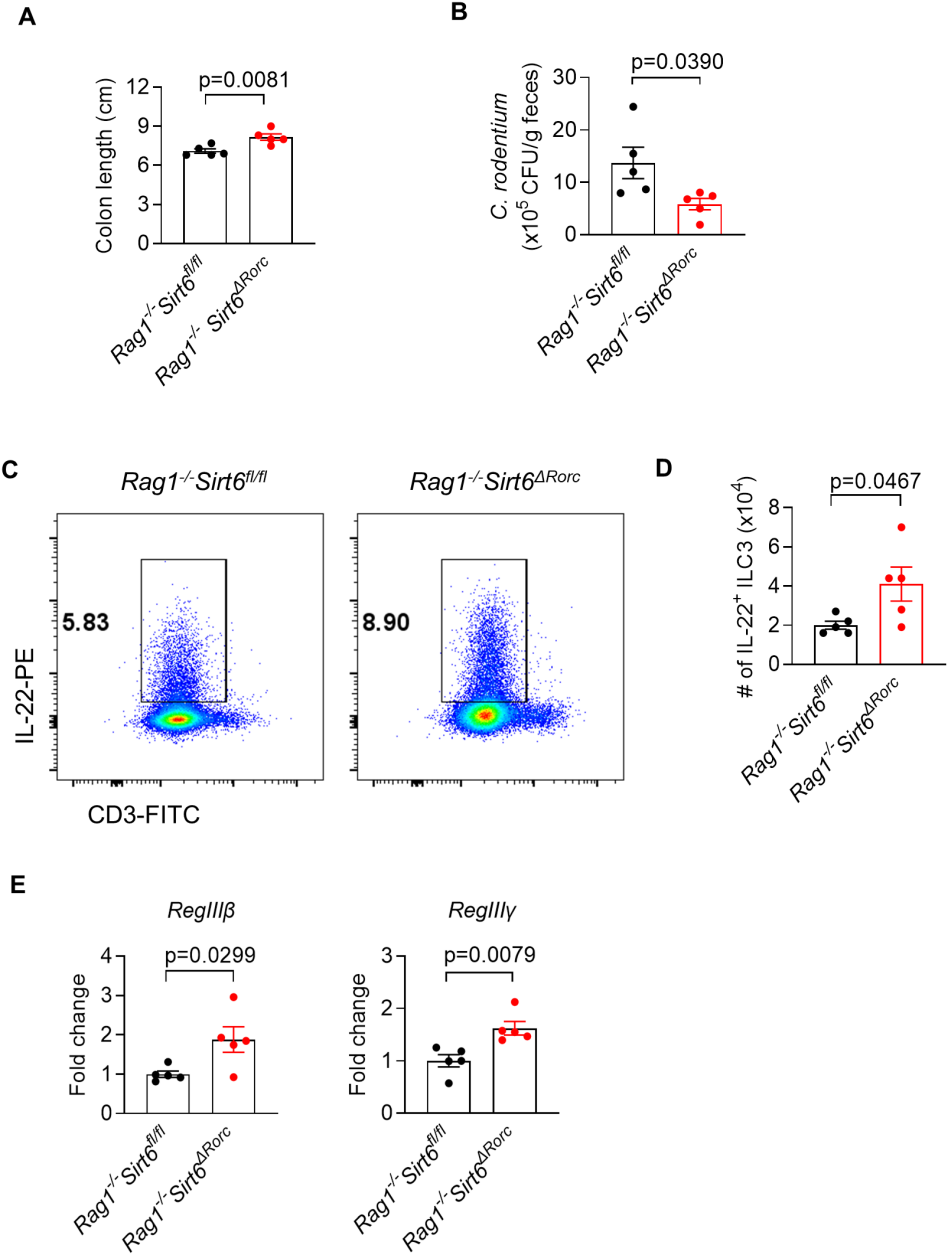
SIRT6 regulates gut immunity against *C. rodentium* infection in an ILC3-intrinsic manner. **(A-E)** *Rag1^-/-^Sirt6^fl/fl^* and *Rag1^-/-^Sirt6^ΔRorc^* mice were infected with *C. rodentium*. Large intestine tissues were collected and analyzed on day 6 post-infection. **(A)** Length of colon and cecum (n=5/group). **(B)** Feces were collected from *Rag1^-/-^Sirt6^fl/fl^* and *Rag1^-/-^Sirt6^ΔRorc^* mice and were used for bacterial load detection. Bacterial counts were normalized to CFU per gram of feces(n=5/group). **(C)** Representative flow cytometry plot of IL-22^+^ ILC3s in large intestine LPLs. Cells were gated on Gr-1^-^B220^-^CD11b^-^CD11c^-^CD45^+^ live lymphocytes. **(D)** Absolute numbers of IL-22 producing ILC3s in large intestine LPLs (n=5/group). **(E)** Analysis of gene expression of *RegIIIβ, RegIIIγ* in large intestine (n=5/group). Each symbol represents an individual mouse **(A, B, D, E)**. Data are representative of 2 independent experiments and are presented as mean ± SEM. For statistical analysis, the following tests were used. A,B,D,E-left, two-tailed unpaired Student’s t-test. E-right, Mann-Whitney test.

To completely rule out the impact of SIRT6-deficient T cells on ILC3s, we crossed *Sirt6^ΔRorc^* mice to *Rag1^−/−^* mice lacking the adaptive immune system. Consistent with the findings in *Sirt6^ΔRorc^* mice, SIRT6-deficient ILC3s had elevated IL-22 production in *Rag1^-/-^Sirt6^ΔRorc^* mice. Next, mice were infected with *C. rodentium* by gavage for 6 days. As expected, *Rag1^-/-^Sirt6^ΔRorc^* mice had longer colon length (**Figure 4A**), reduced bacterial load (**Figure 4B**), increased IL-22-producing ILC3s (**Figures 4C, D**), and enhanced expression of *RegIIIβ, RegIIIγ* (**Figure 4E**). Together, these data support the conclusion that SIRT6 regulates ILC3s in a cell-intrinsic manner during *C. rodentium* infection.

### 3.4 Deletion of SIRT6 in ILC3s protects mice from DSS-induced colitis

**Figure 5:**
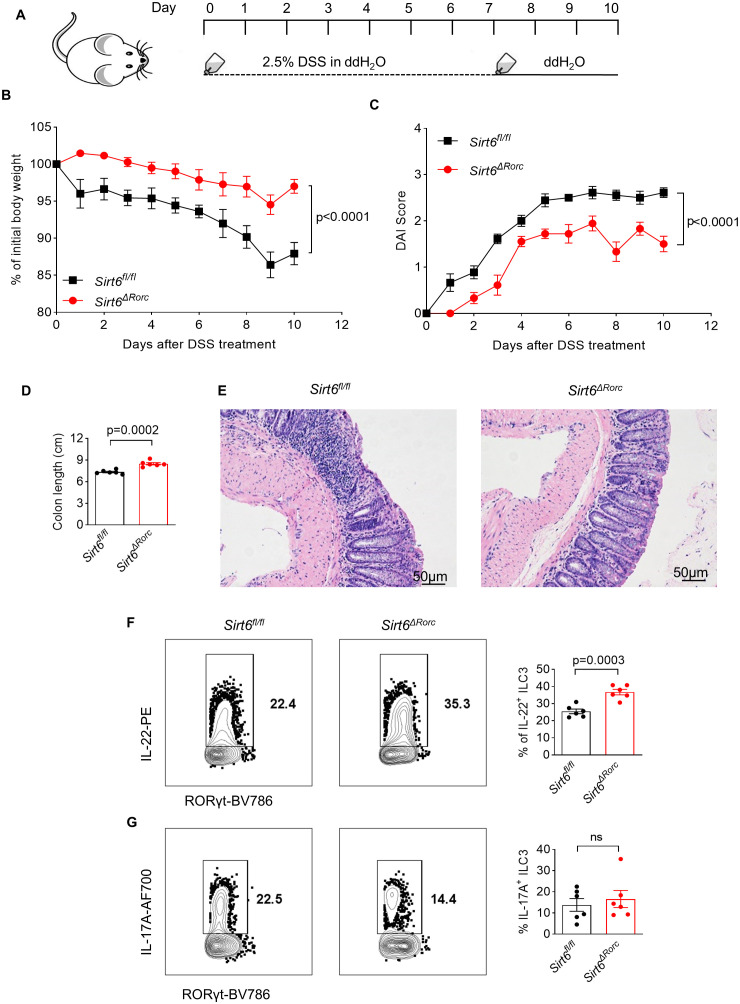
Ablation of SIRT6 in ILC3s protects mice from DSS-induced colitis. **(A)** Experimental scheme for DSS-induced mouse colitis model. *Sirt6^fl/fl^* and *Sirt6^ΔRorc^* mice were given 2.5% DSS in the drinking water for 7 days and then changed to water for another 3 days. The mice were sacrificed on Day 10. **(B)** Weight loss of DSS-treated *Sirt6^fl/fl^* and *Sirt6^ΔRorc^* mice (n=6/group). **(C)** DAI score of DSS-treated *Sirt6^fl/fl^* and *Sirt6^ΔRorc^* mice (n=6/group). **(D)** Colon length of DSS-treated *Sirt6^fl/fl^* and *Sirt6^ΔRorc^* mice (n=6/group). **(E)** Representative image of H&E staining of colon sections from DSS-treated *Sirt6^fl/fl^* and *Sirt6^ΔRorc^* mice at day 10. The scale bar is 50μm. **(F, G)** Representative flow cytometry plots and frequencies of IL-22^+^ ILC3s **(F)** (n=6/group), IL-17A^+^ ILC3s **(G)** (n=6/group) in DSS-treated *Sirt6^fl/fl^* and *Sirt6^ΔRorc^* mice at day10. Cells were gated on live Lin^-^RORγt^+^ lymphocytes. Each symbol represents an individual mouse **(D, F, G)**. Data are representative of 2 independent experiments and are presented as mean ± SEM. For statistical analysis, the following tests were used. B,C, two-way ANOVA. D,F,G, two-tailed unpaired Student’s t-test.

To further determine the role of SIRT6 in regulating ILC3 function during gut inflammation, *Sirt6^ΔRorc^* mice were treated with 2.5% DSS to induce acute colitis (**Figure 5A**). Compared to their littermate controls, *Sirt6^ΔRorc^* mice exhibited less body weight loss, lower DAI scores, and longer colon length (**Figures 5B-D**). Moreover, *Sirt6^ΔRorc^* mice exhibited decreased inflammation, as indicated by fewer infiltrated immune cells in H&E staining (**Figure 5E**). These data suggested that the deletion of *Sirt6* in ILC3s protects mice from DSS-induced colitis. At the cellular level, we observed enhanced IL-22^+^ ILC3s in *Sirt6^ΔRorc^* mice (**Figure 5F**). However, levels of IL-17 were comparable between SIRT6-deficient ILC3s and control ILC3s (**Figure 5G**). Together, these data indicate that SIRT6 negatively regulates ILC3 function during gut inflammation.

### 3.5 SIRT6 inhibitor directly enhances ILC3 function *in vitro*

**Figure 6:**
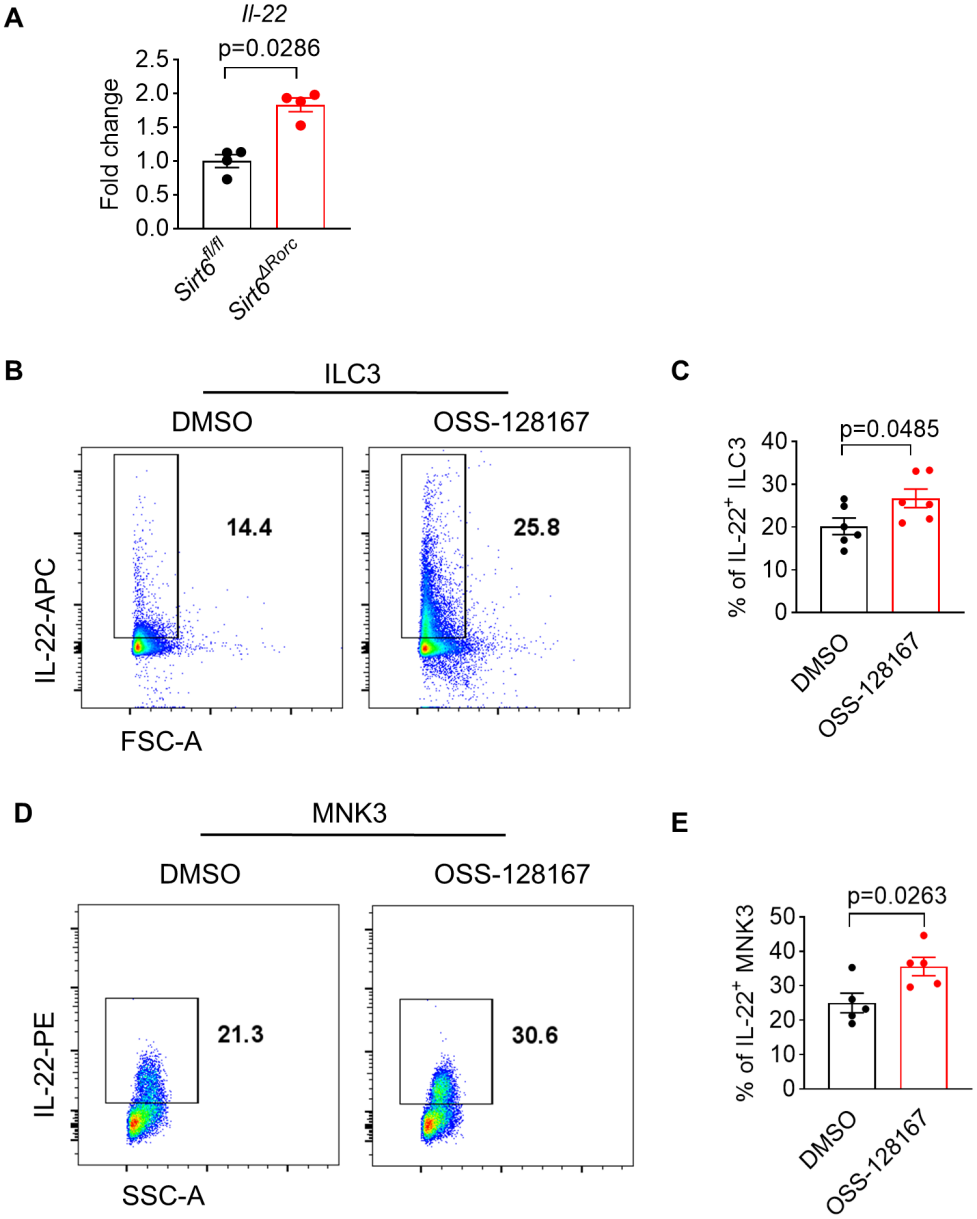
SIRT6 inhibitor directly enhances ILC3 function *in vitro*. **(A)** mRNA expression level of *Il-22* in ILC3s derived from *Sirt6^fl/fl^* and *Sirt6^ΔRorc^* mice (n=4/group). **(B, C)** ILC3s (Lin^-^CD90^hi^CD45^int^) sorted from small intestine were cultured for 18h in the presence of OSS-128167 at 100μM. Representative flow cytometry plots **(B)** and percentage **(C)** of IL-22^+^ ILC3s by flow cytometry (n=6/group). **(D, E)** MNK3 were cultured for 18h in the presence of OSS-128167 at 100μM. Representative flow cytometry plots **(D)** and percentage **(E)** of IL-22^+^ MNK3 by flow cytometry (n=5/group). OSS-128167: SIRT6 inhibitor. Each symbol represents an individual mouse **(A)**. Data are representative of 2 independent experiments and are presented as mean ± SEM. For statistical analysis, the following tests were used. A, Mann-Whitney test. C,E, two-tailed unpaired Student’s t-test.

We also found that the mRNA level of IL-22 was increased in SIRT6-deficient ILC3s, suggesting that SIRT6 regulates IL-22 expression at the transcriptional level (**Figure 6A**). To further investigate whether SIRT6 could directly suppress ILC3 function *in vitro*, a cell-permeable SIRT6-specific inhibitor, OSS-128167, was used to treat ILC3 culture in the presence of IL-23. After treatment with OSS-128167, ILC3s produced more IL-22 (**Figures 6B, C**). In addition, we used the ILC3-like MNK3 cell line, which mimics the phenotype and effector function of murine primary intestinal ILC3s. Similar to the findings observed in ILC3s, *in vitro* OSS-128167 treatment enhanced IL-22 production (**Figures 6D, E**). These data suggest that SIRT6 directly inhibits IL-22 production in ILC3s.

## 4 Discussion

In this study, we have investigated the role of SIRT6 in the regulation of intestinal ILC3 function under steady state and during infection and inflammation. Our data show that SIRT6 is dispensable for the development and differentiation of ILC3, but is required for supporting ILC3 function in a cell-intrinsic manner. We demonstrate that SIRT6 controls ILC3 function by inhibiting IL-22 production, which is critical for maintaining the homeostasis of intestinal immunity. Mice with SIRT6 deficiency in ILC3s are resistant to *C. rodentium* infection and DSS-induced colitis. Thus, SIRT6 acts as a negative regulator of IL-22 production by ILC3s and plays an important role in gut immune defense.

Previous studies have shown that SIRT6 is predominantly expressed in intestinal crypt epithelial cells and plays an important role in regulating intestinal homeostasis (27, 28). Deletion of SIRT6 in IEC results in increased susceptibility of mice to DSS-induced colitis (27). The protective effect of SIRT6 in colitis is considered to act by maintaining the R-sponndin-1 level in the colon (27). Conversely, overexpression of SIRT6 protects mice from DSS-induced colitis due to reduced activation of NF-κB and c-Jun pathways (39). These results indicate that SIRT6 is required to maintain the intestinal barrier function during inflammation. One recent study also shows that SIRT6 can regulate lung epithelial cell function in allergic airway inflammation (29). In addition, SIRT6 can promote intestinal tuft cell development through activation of STAT6, which subsequently enhances intestinal type 2 immune responses to protect mice from helminth infection (28). Therefore, SIRT6 in epithelial cells plays a protective role in gut inflammation and parasite infection. In our study, we found that SIRT6 also regulated gut immunity through modulation of immune cell function. Deletion of SIRT6 in ILC3s resulted in increased IL-22 production and protected mice from *C. rodentium* infection and DSS-induced colitis. However, SIRT6 in ILC3s plays a detrimental role in gut inflammation and pathogenic bacterial infection. These data indicate that SIRT6 plays a differential role in different cell type in the context of gut inflammation. Given that SIRT6 is broadly expressed in multiple cell types in intestinal tissues, it could regulate intestinal homeostasis in a cell-type-specific manner. Thus, targeting SIRT6 need to be considered carefully because it could generate opposite effects in different cell types. It has been shown that SIRT6 expression was decreased in IEC during colitis in both mice and humans (27). However, the downregulation of SIRT6 in ILC3s could promote IL-22 production, leading to enhanced epithelial repair after tissue damage induced by DSS. The enhanced ILC3 function could be explained as a compensatory mechanism to facilitate the repair of epithelial injury.

As a histone deacetylase in cells, SIRT6 can regulate multiple biological functions, including inflammatory gene expression, DNA repair, and aging (24–26). Deacetylation has been linked directly to gene transcription. One study shows that SIRT6 induces RORγt deacetylation in lung epithelial cells, which was subsequently recruited to the IL-17A promoter and increased the transcriptional activity (29). Given that RORγt is also an important transcription factor for IL-22 expression, it raises the possibility that SIRT6 might control IL-22 expression through induction of RORγt deacetylation. However, we did not observe the change of IL-17A expression in SIRT6-deficient ILC3s and SIRT6 inhibited IL-22 expression in ILC3s. These data would rule out the possibility that SIRT6 promotes RORγt transcriptional activity through deacetylation.

In addition, we found that deletion of SIRT6 in RORγt^+^ cells only affected the production of IL-22 in ILC3s, but not in RORγt^+^ T cells. These data suggest that SIRT6 plays a differential role in regulating IL-22 production in ILC3s compared to T cells. As ILC3s sense the tissue environmental signals and produce effector cytokines rapidly, the differences in ILC3s regulated by SIRT6 might be contributed by the distinct activation signals such as IL-23 and IL-1β. This could also be explained by the different cellular metabolism mediated by SIRT6 in ILC3s. Since SIRT6 has been shown to repress the transcriptional activity of HIF1α by deacetylating histone H3K9 at the promoters of several glycolytic genes (40), ILC3s exhibit active mitochondrial metabolism and mROS accumulation, which sustain HIF1α activity to promote IL-22 secretion (35). Given that Th17s have a low rate of mitochondrial oxidative metabolism with little mROS production, the IL-22 expression regulated by HIF1α-mediated metabolic adaption is very unlikely in T cells (41).

This study has some limitations. First, the molecular mechanisms underlying SIRT6 regulation of IL-22 remain unknown. To address this question, RNA sequencing and Cut&Tag of SIRT6-deficient ILC3s need to be done to identify the epigenetic and transcriptional mechanisms. Second, whether the findings we observed in mice can resemble those in humans remains unknown. The expression of SIRT6 in human ILC3s need to be determined. Suppression or activation of SIRT6 with SIRT6 inhibitors or activators on human ILC3 function needs to be investigated as well.

In conclusion, we revealed that SIRT6 acts as a negative regulator of IL-22 production by ILC3s and plays an important role in gut immune defense. These data provided insight into the relation of epigenetic regulators with IL-22 production and supplied a new perspective for a potential strategy against inflammatory bowel disease.

## Data Availability

The original contributions presented in the study are included in the article/**Supplementary Material**. Further inquiries can be directed to the corresponding authors.
