# Supplementary material for: Sirtuin 6 inhibits group 3 innate lymphoid cell function and gut immunity by suppressing IL-22 production
Source: Front Immunol. 2024 Aug 26;15:1402834. doi: 10.3389/fimmu.2024.1402834 (PMC11381250; doi:10.3389/fimmu.2024.1402834)
Supplement: Supplementary file 1 [file DataSheet1.pdf]

Supplementary Material

1 Supplementary Figures and Tables

1.1 Supplementary Figures

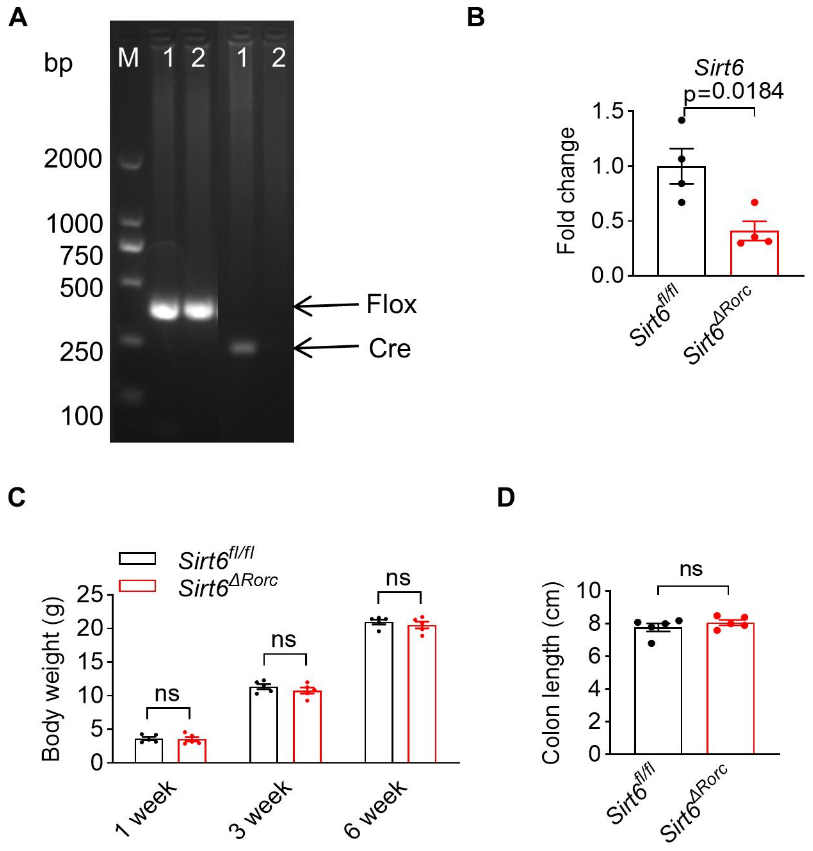

Supplementary Figure 1. Generation of ILC3-specific *Sirt6* knockout (*Sirt6*$^{\Delta Rorc}$) mice. (A) Genotype identification of *Sirt6*$^{fl/fl}$ and *Sirt6*$^{\Delta Rorc}$ mice. (M: DNA marker, 1: *Sirt6*$^{\Delta Rorc}$ mice, 2: *Sirt6*$^{fl/fl}$ mice). **(B)** Expression of *Sirt6* in ILC3s derived from *Sirt6*$^{fl/fl}$ and *Sirt6*$^{\Delta Rorc}$ mice (n=4/group). **(C)** Body weight of *Sirt6*$^{fl/fl}$ and *Sirt6*$^{\Delta Rorc}$ mice(n=5/group). **(D)** Colon length of *Sirt6*$^{fl/fl}$ and *Sirt6*$^{\Delta Rorc}$ mice(n=5/group). Each symbol represents an individual mouse (B-D). Data are representative of 2 independent experiments and are presented as mean ± SEM. Statistical significance was tested by a two-tailed unpaired Student's t-test.

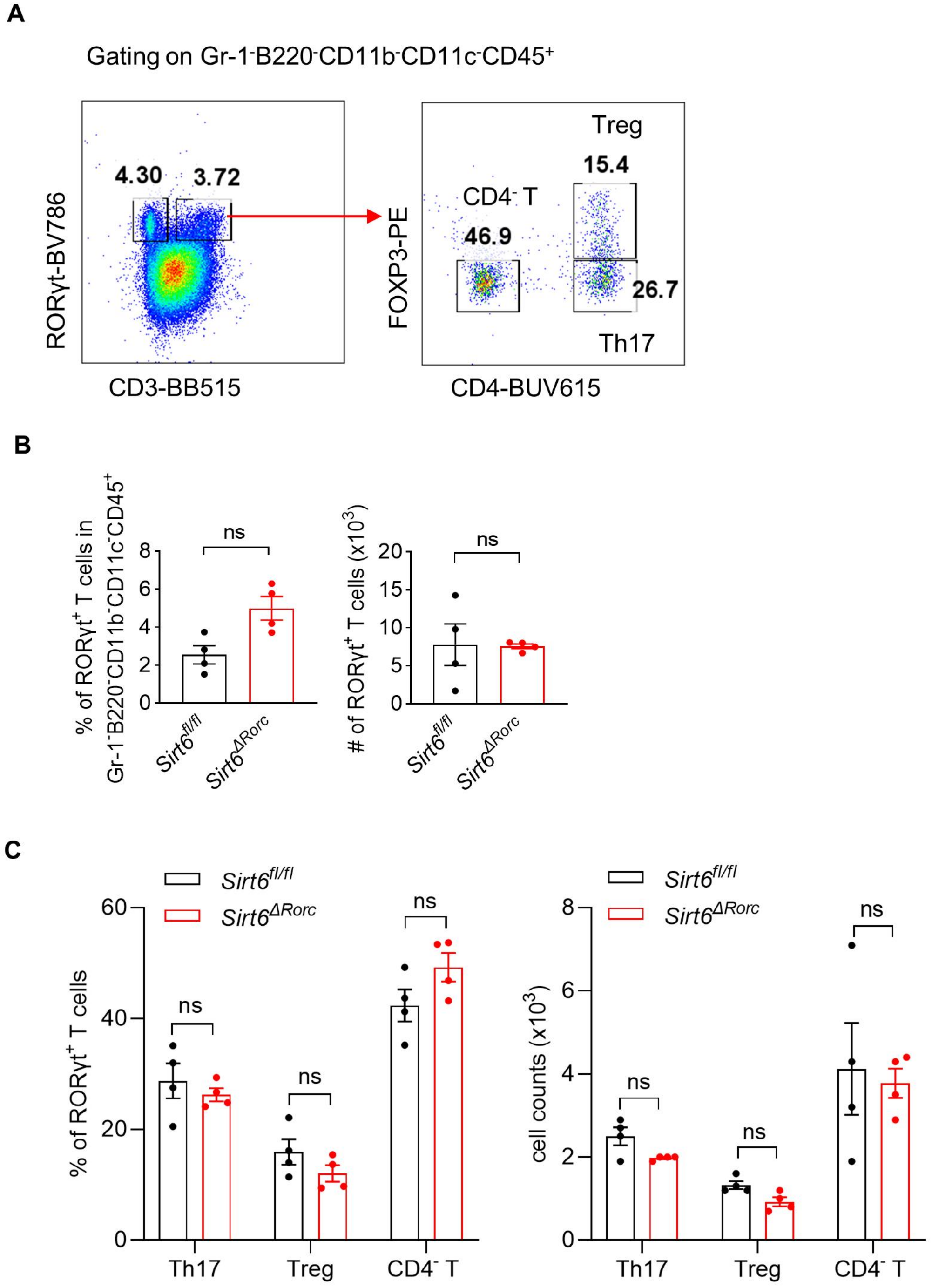

Supplementary Figure 2. RORγt⁺ T cells are not affected in *Sirt6*$^{\Delta Rorc}$ mice. (A) Gating strategy. **(B)** Frequencies and cell count of RORγt⁺ T cells in small intestine lamina propria (n=4/group). **(C)** Frequencies and cell count of RORγt⁺ Th17, RORγt⁺ Treg, and RORγt⁺CD4⁻ T cells (n=4/group). Each symbol represents an individual mouse (B, C). Data are representative of 2 independent experiments and are presented as mean ± SEM. Statistical significance was tested by Mann-Whitney test.

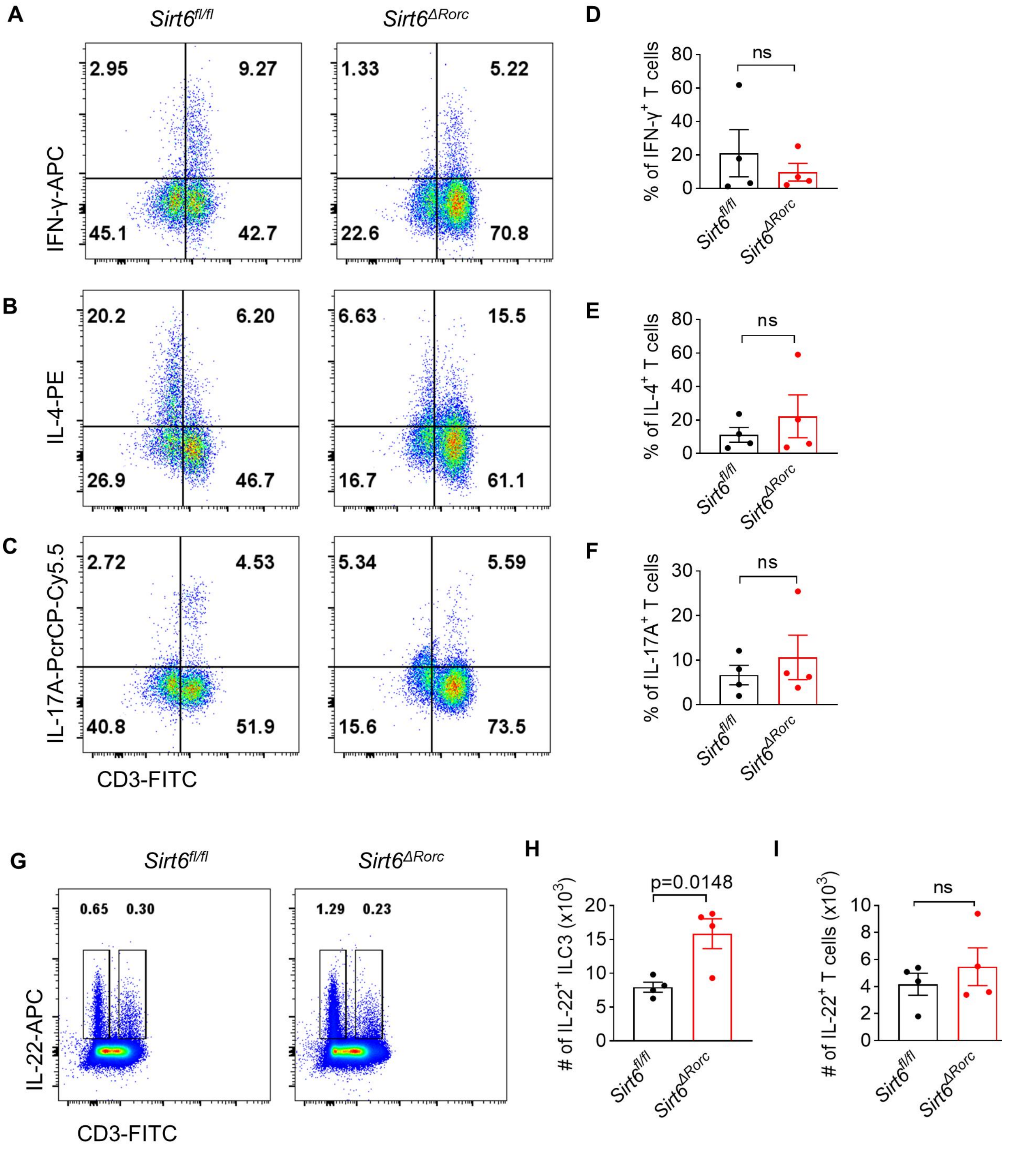

Supplementary Figure 3. Sirt6 deletion does not affect T cell activation. (A-I) LPLs from *Sirt6*$^{fl/fl}$ and *Sirt6*$^{\Delta Rorc}$ mice were treated with PMA and ionomycin for 4h. **(A-C)** Representative intracellular staining for IFN-γ **(A)**, IL-4 **(B)**, and IL-17A **(C)** in small intestine lamina propria CD3$^+$ T cells. Cells was gated on Gr-1$^-$B220$^-$CD11b$^-$CD11c$^-$CD45$^+$ live lymphocytes. **(D-F)** Frequencies of IFN-γ$^+$ T cells **(D)**, IL-4$^+$ T cells **(E)**, and IL-17A$^+$ T cells **(F)**. (n=4/group). **(G)** Representative flow cytometry plot for IL-22 production in ILC3s or T cells. Cells was gated on Gr-1$^-$B220$^-$CD11b$^-$CD11c$^-$CD45$^+$ live lymphocytes. **(H-I)** Absolute count of IL-22$^+$ ILC3s **(H)** and IL-22$^+$ T cells **(I)**. (n=4/group). Each symbol represents an individual mouse (D-F, H-I). Data are representative of 2 independent

experiments and are presented as mean ± SEM. For statistical analysis, the following tests were used. D-F, Mann-Whitney test. H,I, two-tailed unpaired Student's t-test.

1.2 Supplementary Table

Table S1. Reagents used for flow cytometry detection

Reagent	Identifier	Company
Fixable Viability Stain 520	Cat# 564407	BD Biosciences
Fixable Viability Stain 620	Cat# 564996	BD Biosciences
PMA	Cat# P1585-1MG	Sigma-Aldrich
Ionomycin	Cat# ALX-450-007-M001	Enzo Life Sciences
Brefeldin A	Cat# 420601	Biolegend
Recombinant Mouse IL-23	Cat# 1887-ML-010	R&D Systems
Foxp3/Transcription Factor Staining Buffer Set	Cat# 00-5523-00	Thermo Fisher Scientific

Table S2. Antibodies used for flow cytometry

antibody	clone	company
PE-CF594 Hamster Anti-Mouse CD3e	145-2C11	BD Biosciences
PE-CF594 Rat Anti-CD11b	M1/70	BD Biosciences
PE-CF594 Hamster Anti-Mouse CD11c	HL3	BD Biosciences
PE-CF594 Rat Anti-Mouse Ly-6G and Ly-6C	RB6-8C5	BD Biosciences
PE-CF594 Rat Anti-Mouse CD45R	RA3-6B2	BD Biosciences
BV421 Rat Anti-Mouse CD335	29A1.4	BD Biosciences
Alexa 647 Anti-Mouse CD196	140706	BD Biosciences
BV786 Mouse Anti-Mouse ROR γ t	Q31-378	BD Biosciences
Alexa Fluor 700 Rat Anti-Mouse IL-17A	TC11-18H10	BD Biosciences
BUV615 Anti-Mouse CD4	RM4-5	BD Biosciences
TruStain FcX™ (anti-mouse CD16/32) Antibody	93	BioLegend
Alexa Fluor® 700 Anti-mouse CD45.2	104	BioLegend
APC Anti-mouse IL-22	Poly5164	BioLegend
PE Anti-mouse IL-22	Poly5164	BioLegend
FITC Anti-mouse CD3	17A2	BioLegend
PE Anti-mouse IL-4	11B11	BioLegend
PE-Cyanine7 Anti-mouse CD90.2 (Thy-1.2)	53-2.1	eBioscience
FITC Anti-mouse GM-CSF	MP1-22E9	eBioscience

APC Anti-mouse IFN gamma	XMG1.2	eBioscience
PerCP-Cyanine5.5 Anti-mouse IL-17A	eBio17B7	eBioscience
PE Anti-mouse FOXP3	FJK-16s	eBioscience

Table S3. Primers used in this study

Gene	Forward	Reverse
qRT-PCR primers		
Sirt6	CCCGGCTAATGTGGCAGT	CCGTCTACGTTCTGGCTGAC
Il-22	TTGAGGTGTCCAACTTCCAGCA	AGCCGGACGTCTGTGTTGTTA
Il-6	ACCAGAGGAAATTTTCAATAGGC	TGATGCACTTGCAGAAAACA
Il-10	TGAATTCCCTGGGTGAGAAG	TGGCCTTGTAGACACCTTGG
Tnf	CCACCACGCTCTTCTGTCTA	AGGGTCTGGGCCATAGAACT
Il-1β	GCAACTGTTCCTGAACTCAACT	ATCTTTTGGGGTCCGTCAACT
RegIIIβ	ACTCCCTGAAGAATATACCCTCC	CGCTATTGAGCACAGATACGAG
RegIIIγ	ATGCTTCCCCGTATAACCATCA	GGCCATATCTGCATCATACCAG
Hprt	GGGGGCTATAAGTTCTTTGC	TCCAACACTTCGAGAGGTCC
Genotyping primers		
Rorc^{Cre}	TGTCCTGGGCTACCCTACTG	TTCCGGTTATTCAACTTGCAC
Sirt6$^{fl/fl}$	AGTGAGGGGCTAATGGGAAC	AACCCACCTCTCTCCCCTAA
